# Impact of Serum Source on Human Mesenchymal Stem Cell Osteogenic Differentiation in Culture

**DOI:** 10.3390/ijms20205051

**Published:** 2019-10-11

**Authors:** Alexander Popov, Colin Scotchford, David Grant, Virginie Sottile

**Affiliations:** 1 Wolfson Centre for Stem Cells, Tissue Engineering and Modelling (STEM), School of Medicine, University of Nottingham, Nottingham NG7 2RD, UK; alexander.popov3012@gmail.com; 2 Advanced Materials Group, Faculty of Engineering, The University of Nottingham, Nottingham NG7 2RD, UK; colin.scotchford@nottingham.ac.uk (C.S.); david.grant@nottingham.ac.uk (D.G.)

**Keywords:** Mesenchymal stem cells, human serum, fetal calf serum, in vitro differentiation, osteogenic culture

## Abstract

Human mesenchymal stem cells (MSCs) show promise for musculoskeletal repair applications. Animal-derived serum is extensively used for MSC culture as a source of nutrients, extracellular matrix proteins and growth factors. However, the routine use of fetal calf serum (FCS) is not innocuous due to its animal antigens and ill-defined composition, driving the development of alternatives protocols. The present study sought to reduce exposure to FCS via the transient use of human serum. Transient exposure to animal serum had previously proved successful for the osteogenic differentiation of MSCs but had not yet been tested with alternative serum sources. Here, human serum was used to support the proliferation of MSCs, which retained surface marker expression and presented higher alkaline phosphatase activity than those in FCS-based medium. Addition of osteogenic supplements supported strong mineralisation over a 3-week treatment. When limiting serum exposure to the first five days of treatment, MSCs achieved higher differentiation with human serum than with FCS. Finally, human serum analysis revealed significantly higher levels of osteogenic components such as alkaline phosphatase and 25-Hydroxyvitamin D, consistent with the enhanced osteogenic effect. These results indicate that human serum used at the start of the culture offers an efficient replacement for continuous FCS treatment and could enable short-term exposure to patient-derived serum in the future.

## 1. Introduction

Mesenchymal stromal stem cells (MSCs) are able to generate osteoblasts, chondrocytes, and adipocytes [1,2,3]. These progenitors represent prime candidates for therapeutic applications including bone and cartilage regeneration, prevention of graft-versus-host disease, improved engraftment of bone marrow transplants, and the treatment of metabolic diseases [4]. MSCs have primarily been investigated for skeletal repair applications [5] and are one of the few adult stem cell types clinically shown to benefit regenerative approaches. MSC culture in vitro is typically performed in the presence of serum, which is also included for osteogenic differentiation protocols using β-glycerophosphate, ascorbic acid, and dexamethasone supplementation [6].

Serum typically provides nutrients, extracellular matrix proteins, and growth factors to support cells in vitro [7,8]. There is also evidence that serum may act as an antioxidant for cells [9]. Despite its zoogenic content, animal serum has been used since the first isolation of MSCs and remains a prime component for their culture and differentiation. Methods of reducing animal antigens in fetal calf serum (FCS) have been proposed but do not alleviate all risks [10], leading to increasing demands for the development of alternative culture conditions, and a move towards the possible use of human-derived serum [11].

Importantly in the past, transient exposure to FCS has been successful in the osteochondral differentiation of MSCs but has never been expanded to alternative sources of serum [12]. The present study describes an improved alternative approach for the culture and osteogenic differentiation of human MSCs using reduced exposure to serum. Standard FCS-containing medium was compared to osteogenic conditions based on human serum (HS). Different sources of HS were evaluated, and a transient serum regime based on a 5-day human serum exposure before serum-free treatment was then applied to support MSC osteogenic differentiation, over 21 days.

## 2. Results

### 2.1. MSC Culture in HS- or FCS -Based Medium

**Figure 1:**
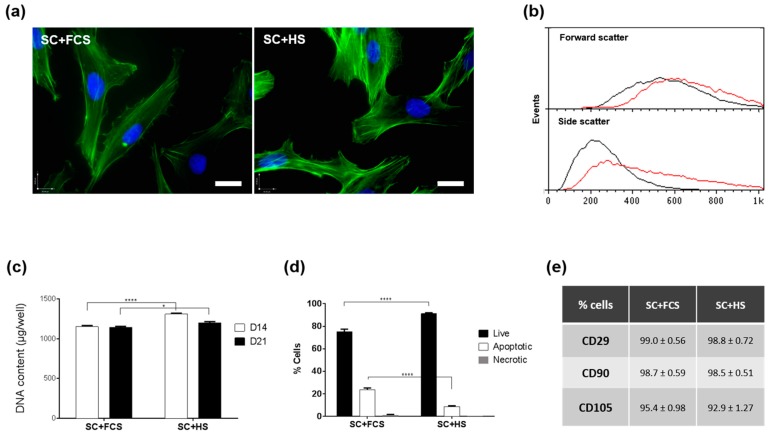
Characterisation of mesenchymal stem cells cultured in fetal calf serum (FCS) and human serum (HS) based medium (SC) over 21 days. (**a**) Fluorescence images of phalloidin-stained MSCs (**green**) cultured in SC+FCS or SC+HS with DAPI nuclear counterstain (**blue**). Scale bar = 50 µm. (**b**) Representative flow cytometry histograms showing the size (Forward scatter, top) and granularity (Side Scatter, bottom) profiles for cultures after two passages in SC+FCS (black) and SC+HS (red). (**c**) DNA quantitation after culture in SC+FCS and SC+HS for 14 (**white**) and 21 days (**black**) (*n* = 6). (**d**) Annexin-V analysis of live, apoptotic and necrotic MSC fractions after 14 days in Standard Culture (SC) medium containing FCS or HS (*n* = 3). * *p* < 0.05 and **** *p* < 0.00005. (**e**) Flow cytometry analysis of MSC surface marker CD29, CD90 and CD105 expression in cells cultured for two passages in SC+FCS or SC+HS. Data presented as mean ± SEM.

In order to evaluate the use of human serum-based medium for MSC cultures, a comparison between the characteristics of cells maintained in fetal calf serum (FCS) or human serum (HS, Lonza) was conducted. MSCs were cultured in either FCS or HS for two passages before their morphology was observed using microscopy (Figure 1). MSCs cultured in both serum types exhibited the typical fibroblastic morphology, however the HS-cultured cells seemed to be more cuboidal in shape than the cells in FCS (Figure 1a). Flow cytometry analysis (Figure 1b) showed no significant difference in cell size between MSCs cultured the two conditions, and the side-scatter data indicated cells cultured in HS to be more granular than cells in FCS, a result supported by bright field microscopy observation that MSCs in HS appeared darker than cells in FCS (data not shown). Cultures in HS-based medium appeared to proliferate at a higher rate than the parallel cultures in FCS-based medium, and DNA content measured at day 14 and day 21 (Figure 1c) was found to be significantly higher in HS culture than FCS culture. The percentage of apoptotic and necrotic cells after 14 days of standard culture medium supplemented with FCS or HS (SC+FCS or SC+HS) determined using Annexin-V staining indicated a higher proportion of apoptotic cells in FCS-culture than cells cultured with HS (Figure 1d). There was also a higher percentage of live MSCs in medium supplemented with HS (91.1%) than medium with FCS (75.3%). Levels of necrosis were negligible for the two conditions. Comparative assessment of CD29, CD90 and CD105 MSC surface marker expression (Figure 1e) indicated analogous levels in cells cultured in HS and cells grown in the presence of FCS.

**Figure 2:**
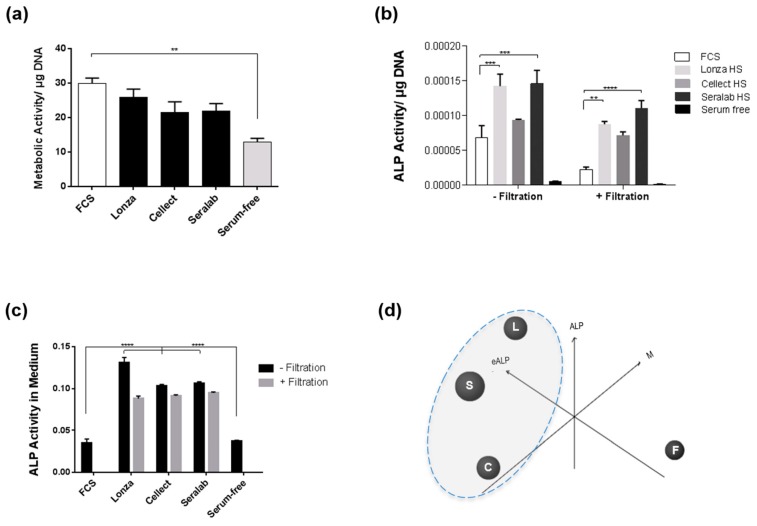
MSC culture using different serum products. (**a**) Metabolic activity in MSCs cultured for five days in SC culture media containing FCS or HS from Lonza, Cellect Seralab, or no serum (serum-free) as controls. (**b**) Alkaline Phosphatase activity for MSCs cultured for five days in SC medium supplemented with FCS or HS sera. (**c**) Alkaline Phosphatase activity measured in the different culture media in the absence of cells. * *p* < 0.05, ** *p* < 0.005, *** *p* < 0.0005, and **** *p* < 0.00005 (*n* = 6). (**d**) 3D plot of cell metabolic assay (M), cell ALP assay (ALP) and endogenous ALP assay (eALP) results for different sera showing clustering of Lonza HS (L), Seralab HS (S) and Cellect HS (C) away from FCS (F).

**Figure A1:**
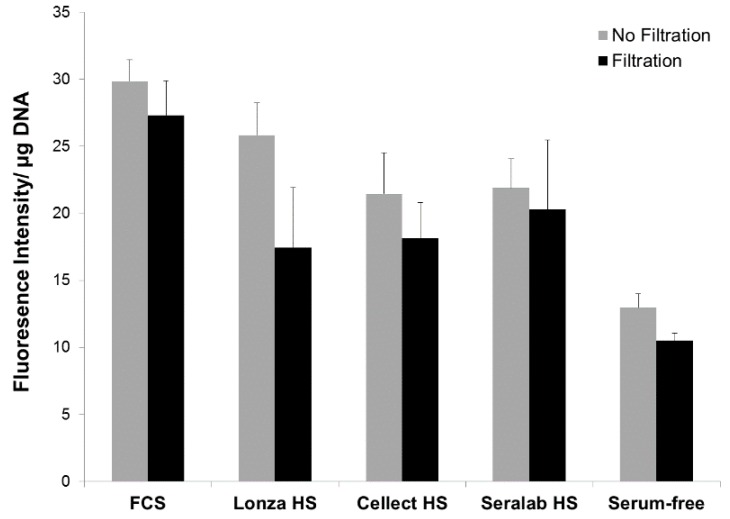
Metabolic activity measured in MSCs cultured for five days in SC medium supplemented with FCS or HS sera, with (black) or without (grey) filtration.

To further investigate whether these effects observed upon exposure to human serum were dependent on reagent source, human sera from three different manufacturers were tested for their effect on metabolic activity and alkaline phosphatase (ALP) activity in culture (Figure 2). Sera from Lonza, Cellect and Seralab were tested in parallel, with serum from Lonza producing the highest cell metabolic activity when compared the Cellect and Seralab products, although this effect was not statistically significant (Figure 2a). Metabolic activity was highest when FCS was used, however levels of ALP activity were significantly higher with HS-based media when compared to FCS-based medium (Figure 2b). Lonza and Seralab sera supported higher ALP activity compared to Cellect, for which results were closer to FCS. Filtration of sera reduced the ALP activity measured for all samples (Figure 2b) while metabolic activity was not significantly affected (Figure A1). To evaluate the contribution of the serum itself to the level of ALP activity measured in the wells, each serum sample was tested for ALP activity in the absence of any cells. HS samples exhibited significantly higher endogenous ALP activity compared to FCS (Figure 2c), which was reduced upon filtration, with the highest reduction observed for Lonza HS (32.8% reduction). Combined analysis of the MSC metabolic assay and ALP activity assay results with the endogenous ALP activity levels measured in each sample showed a clear difference between HS and FCS samples (Figure 2d).

**Figure 3:**
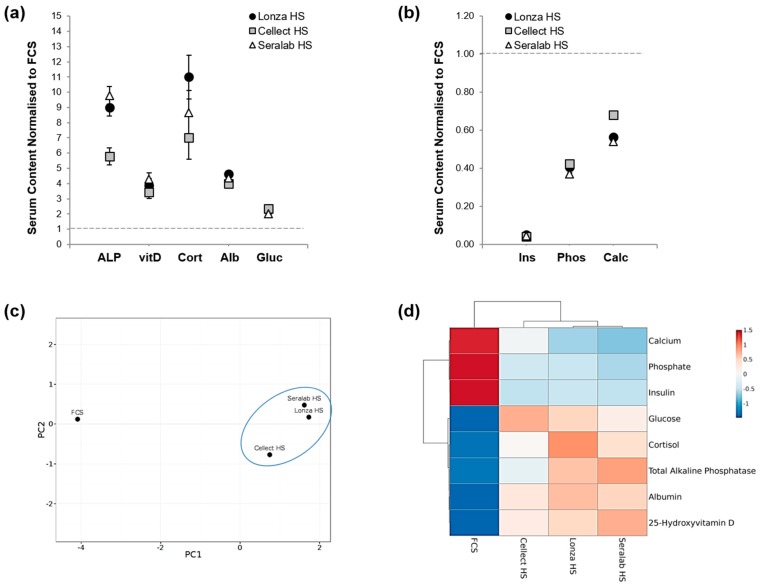
Biochemical analysis of serum components in FCS and HS samples. (**a**) Normalisation to FCS values (dashed line) showed higher HS concentrations for total alkaline phosphatase (ALP), 25-Hydroxyvitamin D (vitD), cortisol (Cort), albumin (Alb), and glucose (Gluc). (**b**) Lower concentrations of insulin (Ins), phosphate (Phos), Calcium (Calc) found in HS samples normalised to FCS (dashed line). * *p* < 0.05, and **** *p* < 0.00005 (*n* = 3). (**c**) Principal component analysis of the different serum samples analysed. (**d**) Heat map of the serum component analysed showing clustering of human samples.

To further investigate the difference between serum conditions used for MSC culture, human sera from Lonza, Cellect and Seralab were biochemically analysed in order to evaluate key component differences compared to fetal calf serum content (Figure 3). Serum analysis showed that levels of glucose, albumin, cortisol, alkaline phosphatase and 1,25-dihydroxyvitamin D were higher in all human serum samples than in fetal calf serum (Figure 3a), while insulin, calcium and phosphate concentrations were conversely higher in FCS (Figure 3b). Although some intra-HS differences were noted as Lonza HS and Seralab HS displayed higher levels of cortisol, alkaline phosphatase, and 25-Hydroxyvitamin D, principal component analysis showed a clear difference between the clustered HS samples and FCS (Figure 3c,d).

### 2.2. Transient Serum Exposure Regimes

**Figure 4:**
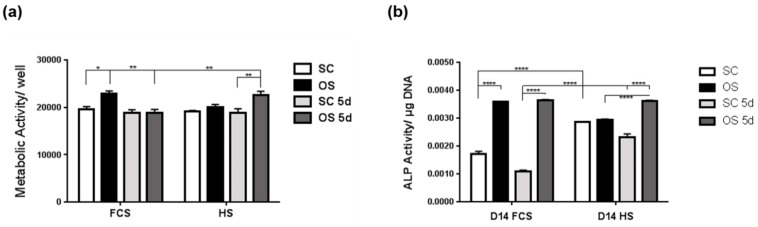
Transient serum exposure for human mesenchymal stem cell cultures with FCS or HS. (**a**) Metabolic activity in MSCs cultured for 14 days in constant (SC, OS) or transient (SC 5d, OS 5d) media using either FCS or HS. (**b**) Alkaline phosphatase activity in MSCs treated for 14 days in standard culture medium (SC) or osteogenic medium (OS) using either constant or transient (5-day) serum exposure with either FCS or HS. * *p* < 0.05, ** *p* < 0.005, and **** *p* < 0.00005 (*n* = 6).

The differentiation potential of MSCs exposed to different serum conditions was analysed using both constant and transient serum exposure to evaluate the dependency of MSC response to FCS or HS. Cultures treated with osteogenic (OS) medium containing human serum from Lonza (OS+HS) or FCS (OS+FCS) throughout the treatment period were compared to cells exposed to transient serum conditions (OS+FCS5d or OS+HS5d), where serum was included for the first five days only before switching to serum-free osteogenic conditions for the rest of the treatment (Figure 4). After 14-day treatments, metabolic activity showed no detectable difference between constant or transient serum exposures for cultures exposed to standard medium (SC) using either serum type (Figure 4a). Under OS conditions however, MSC metabolic activity was differentially affected by transient serum exposure. Reduced FCS exposure led to a decrease in metabolic activity compared to constant FCS medium, while transient HS exposure increased the metabolic response compared to constant HS presence. Measurement of ALP activity in MSCs treated for 14 days demonstrated differences between FCS and HS-containing media. Both constant and transient HS standard media (SC) showed elevated ALP activity when compared to matching FCS conditions. Upon OS treatment, MSC cultures responded to osteogenic conditions by an increase in ALP activity (Figure 4b). For FCS-containing media, transient and constant serum exposure produced similar results, while transient HS presence in the OS treatment led to an increase in ALP activity compared to constant HS (Figure 4b).

**Figure 5:**
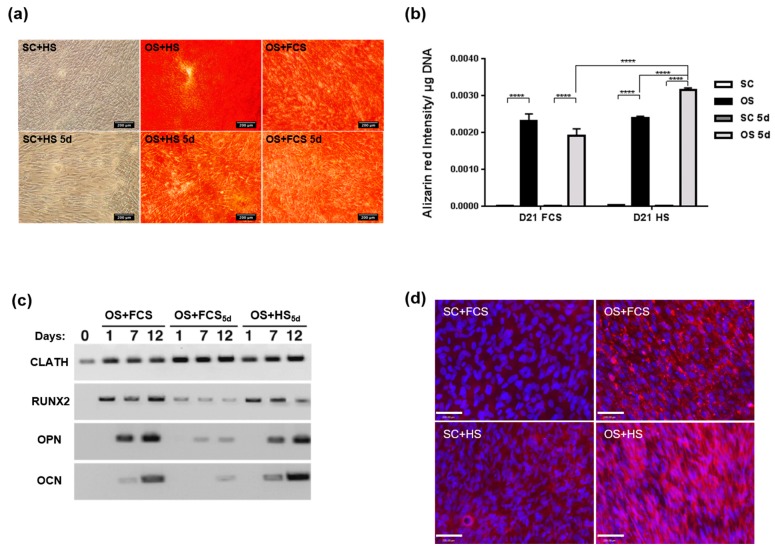
Osteogenic response of human mesenchymal stem cells in the presence of FCS or HS. (**a**) Representative images of Alizarin red staining in MSCs cultures after 21 days in constant and transient HS media compared to constant FCS. (**b**) Quantitation of Alizarin Red staining intensity at day 21. **** *p* < 0.00005 (*n* = 6). (**c**) RT-PCR expression analysis of the osteogenic markers RUNX2, Osteopontin (OPN), and Osteocalcin (OCN) assessed at day one, seven and 12 after the onset of osteogenic treatment. (**d**) Representative images of Osteocalcin immunodetection (**pink**) in MSCs cultured for 21 days in SC or OS media with FCS or HS, with dapi nuclear counterstain (**blue**). Scale bar = 200 μm.

Mineral deposition in MSC cultures increased in response to all tested OS conditions at day 21 (Figure 5a). While transient serum exposure did not have a significant effect when using FCS, it performed better than continuous serum exposure for the HS group (Figure 5b). By day 21, the transient HS treatment group achieved the highest mineral deposition level across all conditions. The osteogenic response of MSCs was further analysed using RT-PCR for the bone-specific genes Runt-related transcription factor 2 (RUNX2), osteopontin (OPN), and osteocalcin (OCN) (Figure 5c). The data showed upregulation of these genes in all OS conditions tested, both in FCS and HS-based media. Transient HS medium showed comparable signal to constant FCS medium, while transient FCS was lower. Upregulation of osteocalcin expression under OS conditions was confirmed by immunocytochemistry (Figure 5d).

### 2.3. Transient Serum Exposure Regimes in Primary MSC Cultures

**Figure 6:**
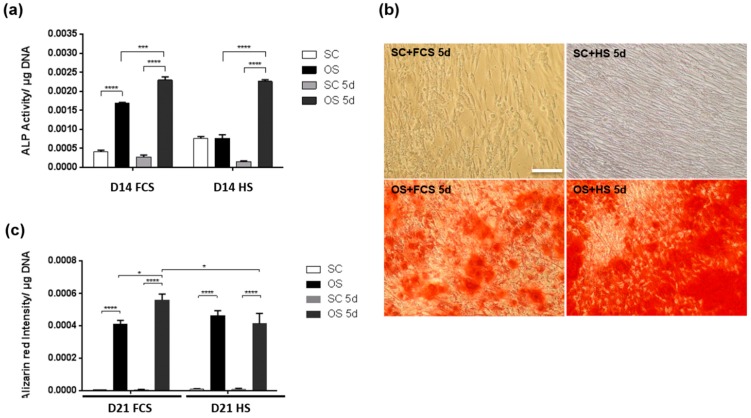
Osteogenic response of primary MSCs in the presence of FCS or HS. (**a**) Alkaline phosphatase activity in MSCs cultured for 21 days in SC, OS, SC 5-Day serum and OS 5-Day serum culture media with FCS or HS. (**b**) Alizarin red staining of MSC cultures after 21 days in SC or OS medium with transient serum exposure using FCS or HS. Scale bar = 200 μm. (**c**) Alizarin red quantification for MSCs cultured for 21 days. * *p* < 0.05, **** *p* < 0.00005 (*n* = 6).

In order to validate the wider efficiency of HS and transient treatments regimes developed with the immortalised cell line, osteogenic differentiation was assessed using primary human MSCs (Figure 6). Similar to data with immortalised MSCs, HS produced higher ALP activity in SC medium compared to FCS. Although primary MSCs demonstrated less osteogenic differentiation than immortalised MSCs, primary cultures responded to differentiation media supplemented with either FCS or HS. Transient serum regimes were also able to trigger this OS response, as measured using an ALP activity assay at day 14 (Figure 6a). At day 21, primary cells cultured in OS conditions with HS appeared to mineralise more than cells in FCS (Figure 6b). However, once mineral deposition was quantified and normalised to DNA content in each well, cells cultured in FCS showed higher levels than HS cells (Figure 6c). Osteogenic cultures treated with transient FCS demonstrated higher ALP activity and more mineral deposition than cells in FCS throughout the differentiation culture period.

## 3. Discussion

### 3.1. Effect of Serum Regimes on Cell Growth

Serum is a critical element of cell culture medium, providing cells with nutrients, ECM proteins and growth factors required for proliferation and differentiation [7]. Although FCS is the most commonly used for the majority of cell systems including MSCs, its use may not be suitable for regenerative therapies because of issues related to animal contaminants even after filtration [1,13]. To evaluate possible HS-based protocols which could lead to autologous serum use [14,15], different HS samples were analysed in the present study, as well as the possibility of limiting the use of HS to the initial treatment phase, a strategy known to successfully support MSC differentiation when applied to FCS medium [12].

When comparing MSCs cultured in SC+FCS or SC+HS media, the typical marker expression and fibroblastic cell morphology were retained, although HS-based cultures appeared more granular and proliferative. This observation is in line with other reports where MSCs were cultured in autologous human serum that was obtained using a completely closed bag system [16]. Others have successfully expanded and differentiated human synovial MSCs using human serum [17].

Cultures in SC+HS exhibited less cell death than in SC+FCS after 14 days, similarly to previous studies using heterologous HS [18], although prolonged heterologous HS use has also been reported to increase growth arrest and death within 6 weeks [1]. Experiments described here were carried out for up to 3 weeks, and therefore further experiments will be required to establish any long-term changes. Our results also support previous observations of higher cell numbers in HS culture compared to FCS [1,16].

A novel strategy to limit the exposure to serum was also tested here, based on a previous study showing that transient FCS treatments can efficiently support MSC growth and differentiation [12] but importantly, this had not thus far been tested with human serum-based approaches. Previous reports have suggested that transient serum exposure may affect MSC growth pattern and morphology [19], while complete serum-free media may not support MSC proliferation [20]. Although, serum-free conditions have been linked to longer telomeres and an increased proliferative capacity [21]. Here, exposure to transient serum in standard culture medium did not lead to reduced metabolic activity when compared to constant serum use.

Although reports have suggested no metabolic differences between FCS and HS during MSC proliferation and differentiation into osteogenic lineages [22], here the response of MSCs to HS and FCS sera was altered under OS medium. FCS (OS+FCS) produced higher metabolic activity than HS (OS+HS) when serum was present throughout, while MSC metabolism was higher with transient HS (OS+HS 5d) than FCS (OS+FCS 5d).

### 3.2. Effect of Serum Regimes on Cell Differentiation

ALP activity, an early osteogenic marker essential in the onset of osteogenic differentiation and mineral deposition [23], was used to evaluate the cellular response in different medium conditions. Human serum produced higher basal level of ALP than FCS in both short- and long-term exposure, even in the absence of any osteogenic supplement. This could be linked to differences in endogenous levels of ALP activity in the bovine and human serum samples.

A report has previously suggested that MSCs expressing high ALP levels in standard conditions do not mineralise in OS medium [24]. This was not supported in this study, as mineralisation was greater in osteogenic cultures with HS than FCS, as was expression of osteocalcin, Runt-related transcription factor 2 (RUNX2) and osteopontin (OPN) [25,26,27].

Significant differences were noted between HS and FCS content, including higher HS levels of known pro-osteogenic factors such as 1,25-dihydroxyvitamin D and cortisol in HS, both linked to the regulation of bone resorption [28]. Vitamin D is known to regulate mineral and skeletal homeostasis in humans and mice [29,30]. Cortisol has been shown to decrease bone formation and bone density via resorption [31], and to promote differentiation in human adipocyte precursor cells in vitro [32].

Higher glucose levels in HS could explain the higher proliferation observed, although there is debate on whether glucose levels can affect growth factor production and proliferation [33]. Levels of calcium, phosphate, two well-documented factors involved in osteogenic differentiation [34,35], were higher in the animal-derived serum. Interestingly, alkaline phosphatase activity in serum can be inhibited by physiological concentrations of phosphate [35], therefore, the lower levels of alkaline phosphatase observed in FCS cultures could be related to higher levels of phosphate. Differences in serum insulin could also be an important factor for osteogenic differentiation of MSCs [36].

The comparative analysis of human serum from three different commercial sources showed consistently higher ALP levels and closer levels of key biochemical compounds when compared to FCS, highlighting possible species differences. The two most pro-osteogenc HS samples (Lonza HS and Seralab HS) shared higher levels of alkaline phosphatase, cortisol and 1,25-dihydroxyvitamin D, all factors cited as important for the osteogenic differentiation of MSCs [29,37]. Future work including a thorough side-by-side comparison of the three HS sources and their cellular effects could provide additional information on the key components driving cell response.

### 3.3. Transient Serum Approaches for Cell Differentiation

Differentiation results with osteogenic medium containing HS for the first five days only were marginally lower than for constant serum culture, showing that the onset of osteogenic differentiation may not be significantly affected in transient HS conditions and contradicting some published data where allogeneic HS resulted in MSC growth arrest and death [38].

Primary mesenchymal stem cells evaluated through Alizarin red staining and ALP quantification showed that transient serum treatments supported differentiation across different cell sources with both FCS and HS [39].

In conclusion, these results show that HS supported MSC growth and differentiation in vitro, offering an alternative to FCS for clinical applications. Rates of osteogenic differentiation were higher using HS compared to FCS culture, an observation shared with other studies considering autologous HS use [1,16]. However, HS is less accessible, more expensive, and more prone to batch variation than FCS, leading to possible limitations for in vitro studies. Furthermore, obtaining sufficient quantities of HS from an autologous donor is difficult [1]. Therefore, the ability of MSCs to grow in transient HS conditions as shown here is of significant importance and suggests that early serum priming is sufficient to achieve in vitro commitment. Further work is needed to compare this approach to serum-deprived models including the use of human platelet lysate [10] or serum-free medium with supplementation of fundamental components present in serum [1] for MSC differentiation.

## 4. Materials and Methods

Reagents were purchased from Thermo Fisher Scientific (UK) unless otherwise stated.

### 4.1. Cell Culture

Immortalised bone marrow-derived human mesenchymal stem cells (MSCs) [40] and primary MSCs (Lonza, Slough, UK) were grown in a humidified atmosphere at 37 °C and 5% CO_2_, in standard culture medium (SC) consisting of low glucose Dulbecco’s Modified Eagle Medium (DMEM), 1% (*v*/*v*) l-Glutamine, 1% (*v*/*v*) non-essential amino acids (NEAA) and 1% (*v*/*v*) antibiotics, supplemented with 10% (*v*/*v*) serum, using either fetal calf serum (SC+FCS) or with human serum (SC+HS) as specified. In a series of targeted experiments, serum exposure was limited to the first five days of culture before withdrawing either FCS (SC+FCS5d) or HS (SC+HS5d) for the reminder of the experiment. MSCs were seeded into 24-well plates at a concentration of 2 × 10^4^ cells/ mL and allowed to attach for 24 h in standard culture (SC+FCS) medium before differentiation was initiated. Osteogenic differentiation of MSCs was initiated using supplementation with dexamethasone (0.1 µM), ascorbic acid phosphate (50 µM), and β-glycerophosphate (10 mM) (Sigma-Aldrich, Gillingham, UK) in one of the following treatments: OS+FCS: osteogenic supplements added to SC+FCS medium; OS+HS: osteogenic supplements added to SC+HS medium; OS+FCS5d: OS+FCS for the first five days before withdrawing the FCS component; OS+HS5d: OS+HS for the first five days before withdrawing the HS component. Six independent replicates were analysed for each condition, and the medium was changed every three days for up to 21 days. In experiments investigating the composition of the sera used, filtration through a 0.2 µm syringe filter (Sartorius, Epsom, UK) was applied, as indicated.

### 4.2. Surface Marker Expression

Cells were harvested using 0.05% trypsin-EDTA, centrifuged at 200× *g* for 5 min, and incubated at room temperature with 5 µL of anti-CD29 (Abcam), -CD90 (eBioscience, Altrincham, UK), -CD105 (AbD Serotec, Altrincham, UK) or -SSEA4 (eBioscience, Altrincham, UK) fluorescently-conjugated antibodies for 30 min. Cells were washed and stored on ice in the dark until analysis using a Beckman Coulter FC500 flow cytometer (Beckman Coulter, High Wycombe, UK). Each surface marker was analysed in triplicates, and the experiment was repeated three times with 50,000 events recorded for each measurement.

### 4.3. Fluorescent Cell Staining

After the culture period, cells were fixed in ice cold 4% paraformaldehyde (PFA) for 15 min and washed in PBS + 0.1% Tween20 (PBT) for 10 min. Fluorescently-tagged phalloidin (Vector Laboratories) was used to image actin filaments following the manufacturer’s instructions. For osteocalcin immunostaining, triplicate samples were blocked for 1 h using 1× Animal-Free Blocker (Vector Laboratories, Peterborough, UK) in PBT. Cells were then exposed to a primary mouse monoclonal anti-osteocalcin antibody (ab13420) (Abcam, Cambridge, UK) dilution of 1:200 in PBT, stored at 4 °C overnight, washed in PBT and incubated with a 1:500 dilution of Texas red-conjugated goat anti-mouse secondary antibody (Vector Laboratories) for 1 h in the dark. After PBS washes, samples were mounted using Vectashield mounting medium containing DAPI (Vector Laboratories) and imaged using a Nikon 90i fluorescence microscope (Nikon, Kingston, UK).

### 4.4. Apoptosis Assay

The percentage of live, apoptotic and necrotic cells after 14 days of osteogenic differentiation with FCS or HS was determined using a commercially available Annexin-V-FLUOS staining kit (Roche, Basel, Switzerland). Manufacturer’s instructions were followed. In brief, cells were washed in PBS, centrifuged at 200× *g* for 5 min and pellets were re-suspended in 100 µL Annexin-V-FLUOS labelling solution. After 15 min of incubation cells were analysed using a Beckman Coulter FC500 flow cytometer.

### 4.5. Cell Growth Measurements

Cell metabolic activity was determined using a PrestoBlue assay. Monolayers were washed with 37 °C PBS. 500 µL of warm working solution (10% *v*/*v* stock solution, 90% *v*/*v* low glucose DMEM) was transferred to each well, with three empty wells filled as blanks, and the plate was incubated at 37 °C, 5% CO_2_ for 35 min. The plate was wrapped in aluminium foil, agitated on a plate shaker for 10 min, and three 100 µL triplicates were analysed for each well using a Tecan Infinite 200 micro-plate reader measuring fluorescence, at 560 nm excitation, 590 nm emission.

Cell proliferation was determined by measuring total DNA content per well over the 21-day culture period. To lyse the cells, the culture medium was replaced with sterile, DNase-free dH_2_O, and the plate was subjected to three freeze-thaw cycles. The stock reagent was diluted 200-fold in 1× dilution buffer. Aliquots of 100 μL from each well were transferred to a 96-well plate, along with 100 μL 1× Quant-iT PicoGreen solution. The plate was gently agitated in the dark for 5 min and analysed using a Tecan Infinite 200 micro-plate fluorescence reader at 480 nm excitation and 520 nm emission. A standard curve of DNA content was produced using known concentrations of lambda DNA.

### 4.6. Alizarin Red Staining and Quantification

After osteogenic treatment, cells were fixed using 4% PFA, washed three times with PBS, and 1 mL of 1% aqueous Alizarin Red solution (Sigma-Aldrich Gillingham, UK) was added per well. The plate was incubated at room temperature for 10 min with occasional agitation, washed with distilled water until all excess stain had been removed, and imaged using a phase contrast microscope [41]. For quantification of Alizarin Red staining, samples (*n* = 6) were incubated with a de-stain solution consisting of 20% methanol, 10% acetic acid in dH_2_O [42] for 15 min with gentle agitation, before the absorbance was measured at 405 nm using a Tecan Infinite 200 micro-plate reader.

### 4.7. Alkaline Phosphatase Assay

Alkaline phosphatase (ALP) enzyme activity was assayed using the SIGMAFAST kit (Sigma-Aldrich, Gillingham, UK) according to the manufacturer’s instructions. Briefly, wells were washed twice with warm PBS, 200 μL of p-Nitrophenyl phosphate substrate solution was added to each well, and the plate was incubated in the dark for 30 min at room temperature, before 100 μL duplicate aliquots/ well were transferred to a 96-well plate. The absorbance for the solutions was read at 405 nm using a Tecan Infinite 200 micro-plate reader. DNA measurements were used to normalise results between conditions.

### 4.8. Serum Biochemical Analysis

Biochemical analyses of serum samples (conducted by the hospital’s Clinical Pathology laboratory at the Queen’s Medical Centre, Nottingham) measured the content of 1,25-dihydroxyvitamin D, albumin, alkaline phosphatase, calcium, cortisol, glucose, insulin and phosphate in triplicates. Principal component analysis was performed using ClustVis (http://biit.cs.ut.ee/clustvis, 2017 version, BIIT, Tartu, Estonia) [43].

### 4.9. RT-PCR

RNA samples were processed as previously described [44] and analysed in triplicates. Samples were PCR-amplified in a Labnet Multigene PCR machine using specific primers (Eurofins MWG) for Osteocalcin (forward: CATGAGAGCCCTCACACTCC, reverse: CAGCAGAGCGACACCCTAGACC), Osteopontin (forward: GACCTGACATCCAGTACCC, reverse: GTTTCAGCACTCTGGTCATC), RUNX2 (forward: CCAGATGGGACTGTGGTTACC, reverse: ACTTGGTGCAGAGTTCAGGG), and Clathrin was used as a housekeeping control.

### 4.10. Statistical Analysis

Statistical analysis was in the form of one-way ANOVA performed using GraphPad PRISM 7 (GraphPad Software, San Diego, CA, USA). Tukey’s post hoc analysis was performed to determine the significance between subgroups of the analysed population. Significance shown as * *p* < 0.05, ** *p* < 0.005, and **** *p* < 0.00005.

## Abbreviations

ALP	Alkaline phosphatase
FCS	Fetal calf serum
HS	Human serum
MSC	Mesenchymal stem cell
PBS	Phosphate-buffered saline
SC	Standard culture
